# Cathepsin K deficiency promotes alveolar bone regeneration by promoting jaw bone marrow mesenchymal stem cells proliferation and differentiation via glycolysis pathway

**DOI:** 10.1111/cpr.13058

**Published:** 2021-05-30

**Authors:** Wuyang Zhang, Zhiwei Dong, Dengke Li, Bei Li, Yuan Liu, Xueni Zheng, Hui Liu, Hongzhi Zhou, Kaijin Hu, Yang Xue

**Affiliations:** ^1^ State Key Laboratory of Military Stomatology & National Clinical Research Center for Oral Diseases & Shaanxi Clinical Research Center for Oral Diseases Department of Oral and Maxillofacial Surgery School of Stomatology The Fourth Military Medical University Xi'an China; ^2^ State Key Laboratory of Military Stomatology Xi'an China; ^3^ State Key Laboratory of Military Stomatology & National Clinical Research Center for Oral Diseases & Shaanxi International Joint Research Center for Oral Diseases Center for Tissue Engineering School of Stomatology The Fourth Military Medical University Xi’an China

**Keywords:** alveolar bone, Cathepsin K, glycolysis, jaw bone marrow mesenchymal stem cells, osteogenic differentiation

## Abstract

**Objectives:**

To clarify the possible role and mechanism of Cathepsin K (CTSK) in alveolar bone regeneration mediated by jaw bone marrow mesenchymal stem cells (JBMMSC).

**Materials and Methods:**

Tooth extraction models of *Ctsk* knockout mice (*Ctsk* ^‐/‐^) and their wildtype (WT) littermates were used to investigate the effect of CTSK on alveolar bone regeneration. The influences of deletion or inhibition of CTSK by odanacatib (ODN) on proliferation and osteogenic differentiation of JBMMSC were assessed by CCK‐8, Western blot and alizarin red staining. To explore the differently expressed genes, RNA from WT and *Ctsk^‐/‐^* JBMMSC was sent to RNA‐seq. ECAR, glucose consumption and lactate production were measured to identify the effect of *Ctsk* deficiency or inhibition on glycolysis. At last, we explored whether *Ctsk* deficiency or inhibition promoted JBMMSC proliferation and osteogenic differentiation through glycolysis.

**Results:**

We found out that *Ctsk* knockout could promote alveolar bone regeneration in vivo. In vitro, we confirmed that both *Ctsk* knockout and inhibition by ODN could promote proliferation of JBMMSC, up‐regulate expression of Runx2 and ALP, and enhance matrix mineralization. RNA‐seq results showed that coding genes of key enzymes in glycolysis were significantly up‐regulated in *Ctsk^‐/‐^* JBMMSC, and *Ctsk* deficiency or inhibition could promote glycolysis in JBMMSC. After blocking glycolysis by 3PO, the effect of *Ctsk* deficiency or inhibition on JBMMSC’s regeneration was blocked subsequently.

**Conclusions:**

Our findings revealed that *Ctsk* knockout or inhibition could promote alveolar bone regeneration by enhancing JBMMSC regeneration via glycolysis. These results shed new lights on the regulatory mechanism of CTSK on bone regeneration.

## Graphical abstract

*Ctsk* knockout or pharmacal inhibition could up‐regulate coding genes of key enzymes in glycolysis including Pfkfb3 and HK1, which promote glycolysis in JBMMSC. Thus, the enhanced glycolysis contribute significantly to the proliferation and osteogenic differentiation of JBMMSC, and finally promote alveolar bone regeneration.

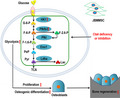

## 1 INTRODUCTION

Cathepsin K (CTSK) is a key enzyme in bone organic matrix degradation. In the beginning, it was thought to be specifically expressed in osteoclasts and played a critical role in bone resorption.^1^, ^2^ In recent years, expression of CTSK in bone formation related cells, such as fibroblasts, osteoblasts and mesenchymal stem cells (MSC), was also confirmed.^3^, ^4^, ^5^ It was found that inhibition of endogenous CTSK could promote the expression of sclerostin in periodontal ligament (PDL) fibroblasts, thereby inhibiting Wnt/β‐Catenin pathway,^4^ indicating that CTSK may be related to osteogenic activity of PDL fibroblasts. However, the exact role of CTSK in these cells remains unclear. Whether CTSK can regulate the proliferation and differentiation of JBMMSC, the most potential seed cells in the field of alveolar bone regeneration,^6^, ^7^ has not been reported, let alone its regulatory mechanism.

Glycocatabolism, the main way to obtain energy, plays a critical role in regulating the regeneration of MSC. It has been reported that hypoxia can increase the pluripotency and self‐renewal of stem cells by enhancing their anaerobic oxidation.^8^, ^9^ It was confirmed that Wnt/LRP5 pathway could promote osteogenic differentiation of ST2 cells by promoting glycolysis,^10^ while canonical Notch pathway could inhibit osteogenic differentiation of BMMSC and ST2 cells by inhibiting glycolysis.^11^ However, there are few reports on the regulation of glycolysis by CTSK.

Yang et al^12^ reported that compared with WT mice, the expression of Glut4 in adipose tissue of *Ctsk^‐/‐^* mice was significantly increased, and glucose metabolism was enhanced. But Dauth et al^13^ found that lack of CTSK in astrocytes appeared not to affect their metabolic supply functions. According to the latest research, selective inhibition of CTSK by ODN can increase the production of reactive oxygen species (ROS) in mitochondria of human renal carcinoma Caki cells, promote mitochondrial fusion and finally enhance tumour cell apoptosis.^14^ However, the role and mechanism of CTSK in regulating glycolysis is still unclear.

In this study, we used tooth extraction models of *Ctsk* ^‐/‐^ and WT littermates to study the role of CTSK in alveolar bone regeneration. We further investigated the effects of *Ctsk* deficiency or pharmacal inhibition on the proliferation and osteogenic differentiation of JBMMSC in vitro. At last, we explored that whether endogenous *Ctsk* deficiency promote JBMMSC regeneration via glycolysis.

## 2 MATERIALS AND METHODS

### 2.1 Animals and tooth extraction

*Ctsk^−/−^* mice were generated by Shanghai Model Organisms Center, Inc (Shanghai, China). Eight‐week‐old *Ctsk^−/−^* mice and their WT littermates were used to extract the bilateral maxillary first molars. At the end of each experimental period (3, 7, 10 and 14 days after tooth extraction), three mice were sacrificed with an excessive dose of anaesthetic to collect their maxillae. All mice were bred and maintained in the SPF Laboratory Animal Center of the Fourth Military Medical University. This study was carried out in accordance with the Institutional Animal Care Guidelines and approved by the Laboratory Animal Care & Welfare Committee, School of Stomatology, Fourth Military Medical University (Approval ID 2020‐063).

### 2.2 Micro‐computed tomography

All maxillae were fixed in 4% paraformaldehyde. Next, all specimens were scanned by Micro‐computed tomography (Micro‐CT) (Siemens Inveon Micro‐CT, Siemens AG) and performed at a voltage of 80 kV, a current of 500 μA and a resolution of 10 μm. Subsequently, three‐dimensional images were reconstructed using the Inveon Research Workplace (Siemens AG). The region of interest included the whole three tooth extraction sockets. The morphological parameters of trabecular bone microarchitecture, including bone volume fraction (BV/TV, %), trabecular thickness (Tb. Th, mm) and trabecular separation (Tb. Sp, mm), were measured.

### 2.3 Histology and histochemistry staining

All specimens were demineralized in 10% EDTA (pH 7.4) for 4 weeks, and 5 μm sections of paraffin‐embedded maxillae were obtained. The paraffin sections were stained with haematoxylin and eosin (H&E), histochemically for tartrate‐resistant acid phosphatase (TRAP) (Sigma, Cat#387A) and Masson’s trichrome staining (Solarbio, Cat#G1345) according to the manufacturer's instructions. Osteoclasts were defined as multinuclear TRAP‐positive cells, and the number of osteoclasts per bone surface (N. Oc/BS) of the distobuccal socket was determined.

### 2.4 Immunohistochemistry staining

For immunohistochemistry, slides were preincubated with 3% H_2_O_2_ for 10 minutes. Goat serum was used to block the nonspecific binding, and then, sections were incubated overnight at 4°C with rabbit anti‐cathepsin K polyclonal antibody (Abcam, Cat#ab19027) or rabbit anti‐Osx polyclonal antibody (Abcam, Cat#ab209484). Subsequently, sections were incubated with horseradish peroxidase‐conjugated secondary antibody for 30 minutes at 37°C. Colour was developed using DAB substrate kit (Boster, Cat#13J25J14J1022), and haematoxylin was used for counterstaining. The number of positive cells per unit area in the distal socket was calculated.

For immunofluorescence staining, the slices were blocked with 5% BSA and then incubated overnight at 4°C with a rat anti‐CD90 polyclonal antibody (BioLegend, Cat# 105201), a rat anti‐CD44 polyclonal antibody (BioLegend, Cat#103003), goat anti‐Osx monoclonal antibody (Santa Cruz, Cat# sc‐393325) and a rabbit anti‐CTSK polyclonal antibody (Abcam, Cat#ab19027). After that, the sections were incubated with anti‐rat IgG (H + L) (Proteintech, Cat# SA00003‐11), donkey anti‐goat IgG (H + L) (R&D, Cat#F0108) and goat anti‐rabbit IgG (H + L) (Proteintech, Cat#SA00013‐2) for 30 minutes. Nuclei were counterstained with DAPI. Images were captured on a Nikon A1plus confocal laser scanning microscope.

### 2.5 Isolation, culture and identification of JBMMSC

Mandibles from 6‐week‐old WT and *Ctsk^−/−^* mice were separated. The attached soft tissues, teeth and cartilage were removed. Then, the mandibular bones were washed with PBS with penicillin/streptomycin and cutted into 1 mm^3^. Then, shredded tissues were seeded in culture dishes and cultured in α‐MEM (Hyclone, Cat#SH30265.01) supplemented with 20% foetal bovine serum (Hyclone, Cat#SH30406.05). The medium was changed every 2 days. Cells of passage 2 were used for the subsequent experiments. For identification of JBMMSC, flow cytometry was used to detect the positive surface markers: CD90 (BioLegend, Cat#150306), CD105 (BioLegend, Cat#120408) and CD44 (BioLegend, Cat#103006) and negative surface markers: CD45 (BioLegend, Cat#103108) and CD34 (BioLegend, Cat#128610).

### 2.6 Immunocytochemistry

The JBMMSC were seeded on the glass slides until 80% confluence. Then Lyso Tracker (KeyGEN, Cat#KGMP006) was added and incubated with cells at 37°C for 1 hour. After that, cells were fixed in 4% paraformaldehyde. Cells were permeabilized in PBS containing 0.5% Triton X‐100 for 20 minutes and blocked with normal goat serum for 30 minutes at room temperature. Then, the cells were incubated overnight at 4°C with a rabbit anti‐CTSK polyclonal antibody (Abcam, Cat#ab19027). After that, the sections were incubated with goat anti‐rabbit IgG (H + L) (Proteintech, Cat#SA0013‐2) at 37°C for 30 minutes. Nuclei were counterstained with DAPI.

### 2.7 Osteogenic differentiation

Jaw bone marrow mesenchymal stem cells were cultured under osteogenic culture conditions in medium containing DMEM complete medium, 10 mmol/L β‐glycerol phosphate, 50 μmol/L ascorbate and 10^−7^ M dexamethasone (Cyagen, Cat#MUBMX‐90021). The medium was changed every 3 days. After osteogenic induction for 7 days, Western blot was performed to analyse the osteogenesis‐related proteins (ALP and Runx2). Fifteen days after induction, alizarin red staining was used to assess matrix mineralization.

### 2.8 Cell proliferation assay

For cell proliferation assay, cells were seeded into 96‐well plates at a density of 4 × 10^3^ cells/well, and then the cells were treated with 1 μmol/L ODN (MCE, Cat# HY‐10042), 1 μmol/L ODN + 10 μmol/L 3PO (MCE, Cat#HY‐19824), 10 μmol/L 3PO or 1 μmol/L DMSO. Then, cell proliferation was estimated by CCK‐8 kit (EnoGene, Cat#E1CK‐000208) according to the manufacturer’s instructions. Briefly, cells were incubated with 10 μL CCK‐8 solutions for 90 minutes at 37°C, and then, absorbance of each well at 450 nm was recorded.

### 2.9 Western blot analysis

Cells were lysed with RIPA lysis buffer at 4°C for 30 minutes. Twenty micrograms of protein was separated by SDS‐PAGE and transferred to PVDF membranes. Then, membranes were blocked with 5% BSA in PBST for 1 hour. All membranes were incubated overnight at 4°C with the following primary antibodies: anti‐β‐actin (Proteintech, Cat#20536‐1‐AP), anti‐CTSK (Abcam, Cat#ab19027), anti‐ALP (Abcam, Cat#ab65834), anti‐Runx2 (Abcam, Cat#ab23981). Then, the membranes were incubated with secondary antibody (Abcam, Cat# ab6721) diluted at 1:5000 in PBST. The protein bands were detected using an enhanced chemiluminescence kit (Proandy, Cat# 10144). The quantification of Western blot was analysed using Image J (National Institutes of Health).

### 2.10 RNA‐seq and bioinformatics analysis

Total RNA was extracted from WT and *Ctsk^‐/‐^* JBMMSC and subjected to RNA sequencing by Beijing Genomics Institute (ShenZhen, China). In brief, mRNA sequencing was performed using BGISEQ‐500 platform and the high‐quality reads were aligned to the mouse reference genome (GRCm38). Gene expression was established by the number of fragments per kilobase of exon per million fragments mapped reads by Expectation Maximization. Differentially expressed genes were defined by both the fold change (FC ≥ 1.2) and statistical difference (*P* < .05).

### 2.11 Quantitative real‐time PCR (RT‐qPCR)

Total RNA was extracted from cells with the TRIzol reagent™ (Invitrogen) according to the manufacturer's instructions. cDNA was synthesized using PrimeScript™ RT Master Mix (Takara, Cat# RR036A). RT‐qPCR was performed by TB Premix Ex Taq™ II kit (Takara, Cat# RR820A) and then detected on the CFX96 Real‐Time System (Bio‐Rad). The mRNA levels were calculated using $2^{‐\Delta \Delta Ct}$ method after normalization to the expression of β‐actin. The primers were described in Table S1

### 2.12 Extracellular acidification rate

Cells were plated at 4 × 10^4^ cells/well in a Seahorse XF24 Cell Culture Microplate (Agilent). Twenty‐four hours later, WT cells were treated with 1 μmol/L ODN or 1 μmol/L DMSO for 48 hours. After that, the medium was switched to Seahorse XF Base Medium (Seahorse, Cat#103575‐100) supplemented with glutamine and then incubated for 1 hour in a non‐CO_2_ incubator at 37°C. Cells were treated with 10 mmol/L glucose, 1 μmol/L oligomycin and 50 mmol/L 2‐DG. Then extracellular acidification rate (ECAR) were measured with an XFe96 extracellular flux analyser (Seahorse Bioscience).

### 2.13 Glucose consumption and lactate production

For glucose consumption measurements, cells were seeded into six‐well plates at a density of 2 × 10^5^ cells/well. Twenty‐four hours later, WT cells were treated with 1 μmol/L ODN or 1 μmol/L DMSO for 48 hours. Supernatants were collected to analyse the level of glucose by Glucose (HK) Assay Kit (Sigma, Cat#GAHK20) according to manufacturer's instructions.

For lactate production, cells were seeded into T25 culture flask at a density of 8 × 10^5^ cells/flask. Twenty‐four hours later, WT cells were treated with 1 μmol/L ODN or 1 μmol/L DMSO for 48 hours. Cells were collected to analyse the production of lactate by l‐Lactate Assay kit (Abcam, Cat# ab65331) according to manufacturer’s instructions.

### 2.14 Statistical analysis

All quantitative data were presented as means ± SD. Statistical analysis was performed using GraphPad Prism Software. Comparisons between two groups were analysed by two‐tailed, unpaired Student’s *t* tests. Comparisons between more than two groups were performed by one‐way ANOVA. Statistical significance was defined as: *P < .05, **P < .01, ***P <.001.

## 3 RESULTS

### 3.1 *Ctsk* deficiency promotes alveolar bone regeneration

**FIGURE 1:**
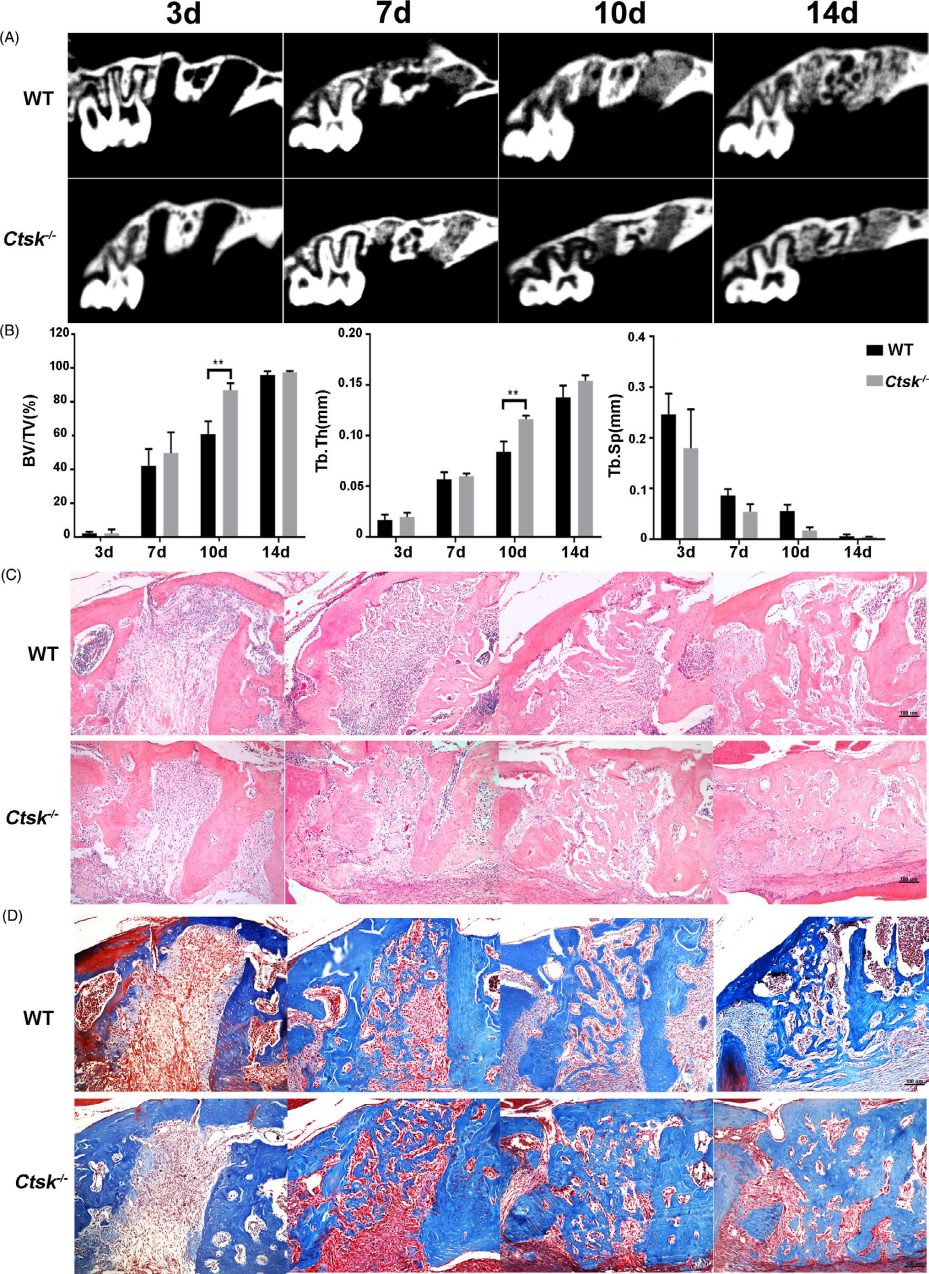
*Ctsk* deficiency promotes alveolar bone regeneration. Eight‐week‐old *Ctsk^−/−^* mice and their WT littermates were used for tooth extraction. Three mice were sacrificed 3, 7, 10 and 14 d after tooth extraction. A, Representative Micro‐CT scanning images of the extraction socket along the longitudinal direction of the maxillae. B, BV/TV (%), Tb. Th (mm) and Tb. Sp (mm) of trabecular bone in the extraction socket were analysed. C, Representative images of H&E staining paraffin sections. D, Representative images of Masson’s trichrome staining paraffin sections. The statistical analysis was shown: **P < .01

To explore the role of CTSK in alveolar bone regeneration, 8‐week‐old *Ctsk* ^‐/‐^ mice and their WT littermates were sacrificed 3, 7, 10 and 14 days after tooth extraction. Then, Micro‐CT scanning, H&E staining and Masson’s trichrome staining were performed. Micro‐CT and histomorphometric analyses revealed that the new bone formation began at the bottom of socket on D7, and then, the new bone gradually expanded in the course of time and filled the entire socket on D14. Importantly, more bone was formed in *Ctsk* ^‐/‐^ mice compared with that in WT mice on D10 (Figure 1A), both BV/TV and Tb. Th were significantly increased in *Ctsk* ^‐/‐^ mice compared with that in WT mice (*P* <.05) (Figure 1B).

In addition, histomorphometric analysis of both H&E‐stained and Masson’s trichrome stained tissue sections (Figure 1C,D) showed that on D3, most of the fibres were located in the residual PDL in WT mice, while a small amount of fibres could be found at the bottom of the socket in *Ctsk* ^‐/‐^ mice. In another word, bone regeneration in the extraction socket was initiated earlier in *Ctsk* ^‐/‐^ mice. On D10, more new trabeculae could be seen in *Ctsk* ^‐/‐^ mice. However, on D14, the form and structure of most trabeculae were organized in both WT and *Ctsk* ^‐/‐^ mice. In general, *Ctsk* deficiency could promote early bone formation after tooth extraction.

### 3.2 *Ctsk* deficiency accelerates osteoblast activity during the process of alveolar bone filling

**FIGURE 2:**
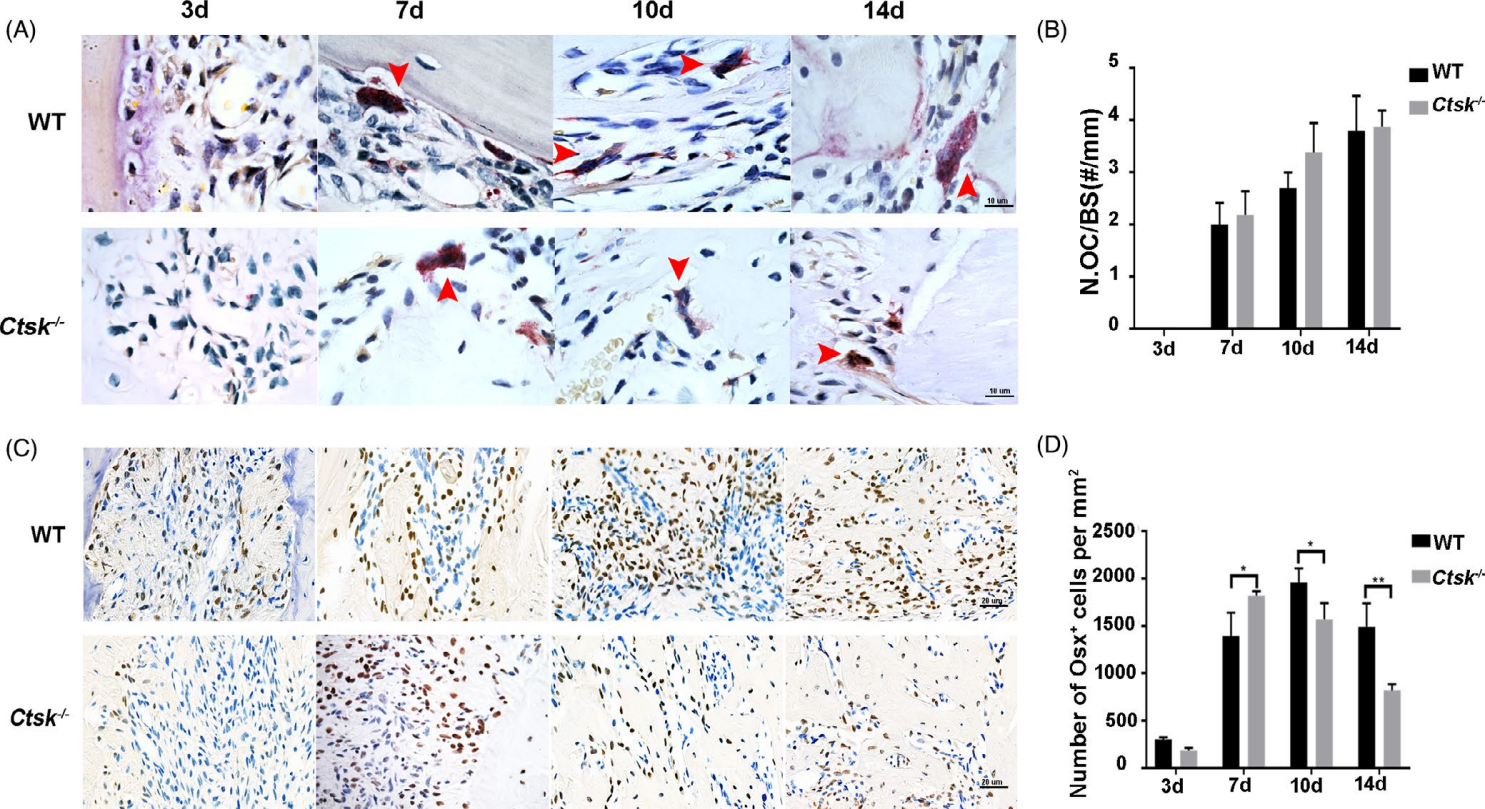
*Ctsk* deficiency accelerates osteoblast activity during the process of alveolar bone filling. A, Representative images of TRAP‐stained paraffin sections. Red arrowheads indicate osteoclasts. B, Number of TRAP‐positive osteoclast per alveolar bone surface was analysed. C, Immunohistochemistry staining of osterix in extraction socket healing process. D, Quantitative analysis of Osx‐positive cells. The statistical analysis was shown: *P < .05, **P < .01

There are two processes, including bone filling and bone remodelling, after tooth extraction. In mice, the bone filling stage was during the first 14 days, while the bone remodelling stage began with the onset of bone filling and lasted for 35 days, with an extremely active stage from D14 to D21 (Figure S1). During the bone filling stage, osteogenesis was dominant, while a small number of osteoclasts (2‐4/mm) could also be seen on the surface of new bone (Figure 2A). However, there was no significant difference in the number of osteoclasts between *Ctsk* ^‐/‐^ and WT mice (Figure 2B). To investigate whether *Ctsk* deficiency could promote alveolar bone regeneration through affecting osteogenic capability, immunohistochemistry was used to study the expression of osterix (Osx), which is an essential transcription factor for osteoblast differentiation and bone formation during the alveolar bone regeneration process. In both *Ctsk^‐/‐^* and WT mice, a small amount of Osx‐positive cells (recognized as osteoblasts) could be observed at the bottom of the socket before new bone formation (Figure 2C). The number of Osx‐positive cells increased significantly as more and more new bone formed. On the contrary, the number of Osx‐positive cells began to decrease when the whole socket was filled. Because it took 10 days for the extraction sockets to be full filled in *Ctsk^‐/‐^* mice and 14 days in WT mice, the number of Osx‐positive cells in *Ctsk^‐/‐^* mice peaked on D7, while that in WT mice peaked on D10 (Figure 2D). These findings indicated that *Ctsk* knockout could accelerate the osteogenic capability during alveolar bone filling process.

### 3.3 CTSK is expressed in MSC‐like cells and osteoblasts during the process of alveolar bone filling

**FIGURE 3:**
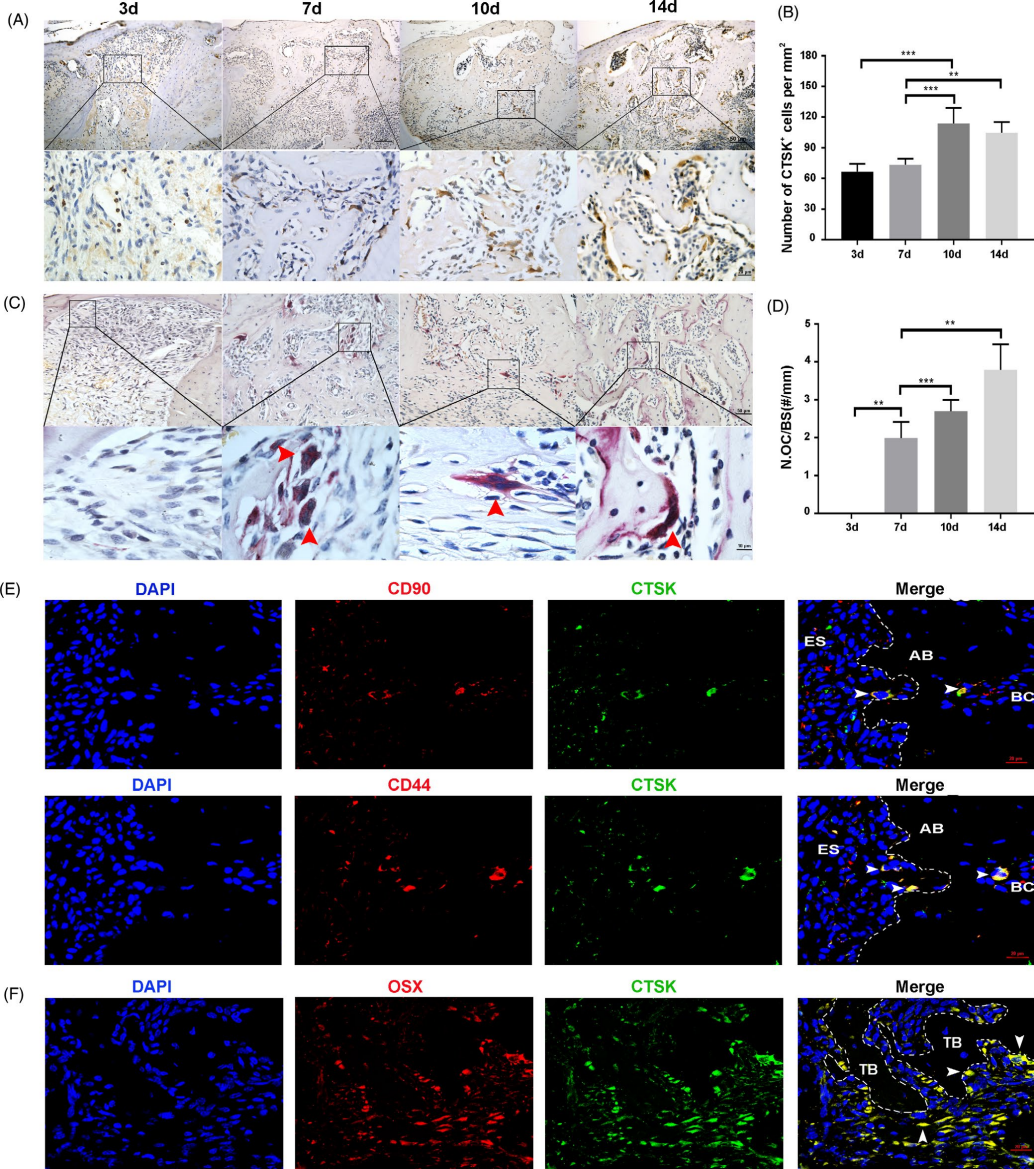
CTSK is expressed in JBMMSC and osteoblasts during the process of alveolar bone filling. A, Immunohistochemistry staining of CTSK in the extraction socket filling process. B, CTSK‐positive cells per mm^2^ was assessed. C, Distribution of osteoclasts was analysed by TRAP staining. Red arrowheads indicate osteoclasts. D, Number of TRAP‐positive osteoclast per alveolar bone surface was analysed. E, Immunofluorescent staining of CTSK (green) in extraction socket (7d post‐extraction). CD44 (red) and CD90 (red) were stained as the bone marrow mesenchymal stem cells marker. Nuclei were stained with the DAPI (blue). White arrowheads indicate CTSK^+^/CD90^+^ or CTSK^+^/CD44^+^ cells. F, Immunofluorescent staining of CTSK (green) and Osterix (red) in extraction socket (7d post‐extraction). Nuclei were stained with the DAPI (blue). White arrowheads indicate CTSK^+^/OSX^+^cells. The statistical analysis was shown: **P <.01; ***P <.001. AB, alveolar bone; BC, bone marrow cavity; ES, extraction socket; TB, trabecular bone

To explore how *Ctsk* deficiency accelerates the osteogenic capability during the process of alveolar bone filling, the expression of CTSK during alveolar bone regeneration was detected. The results of immunohistochemistry staining of CTSK and TRAP staining revealed that as a key molecule involved in bone resorption, the distribution of CTSK (Figure 3A,B) differed from that of osteoclasts (Figure 3C,D) in the filling process. CTSK‐positive staining was observed on D3, when neither osteoclasts nor newly formed bone was observed. At this point, CTSK was mainly observed in some fibroblast‐like cells in the granulation tissues and residual periodontal membrane. On D7, a few osteoclasts appeared with the beginning of new bone formation. Strong staining of CTSK was observed in osteoclasts, lymphocyte‐like cells and osteoblast‐like cells. The most impressively, CTSK positivity was also observed in a few CD44^+^/CD90^+^ and Osx^+^ cells located on the surface of the alveolar bone (Figure 3E,F). On D10 and D14, the number of CTSK‐positive cells peaked; strong staining of CTSK was observed in both osteoclasts and osteoblast‐like cells located on the surface of the newly formed bone in the socket. During D21 to D35, CTSK was mainly expressed in osteoclasts. The number of CTSK‐positive cells was similar to that of osteoclasts (Figure S2). To sum up, during the process of alveolar bone filling, CTSK was expressed not only in osteoclasts, but also in MSC‐like cells and osteoblasts.

### 3.4 Endogenous *Ctsk* deficiency promotes JBMMSC proliferation and osteogenic differentiation

**FIGURE 4:**
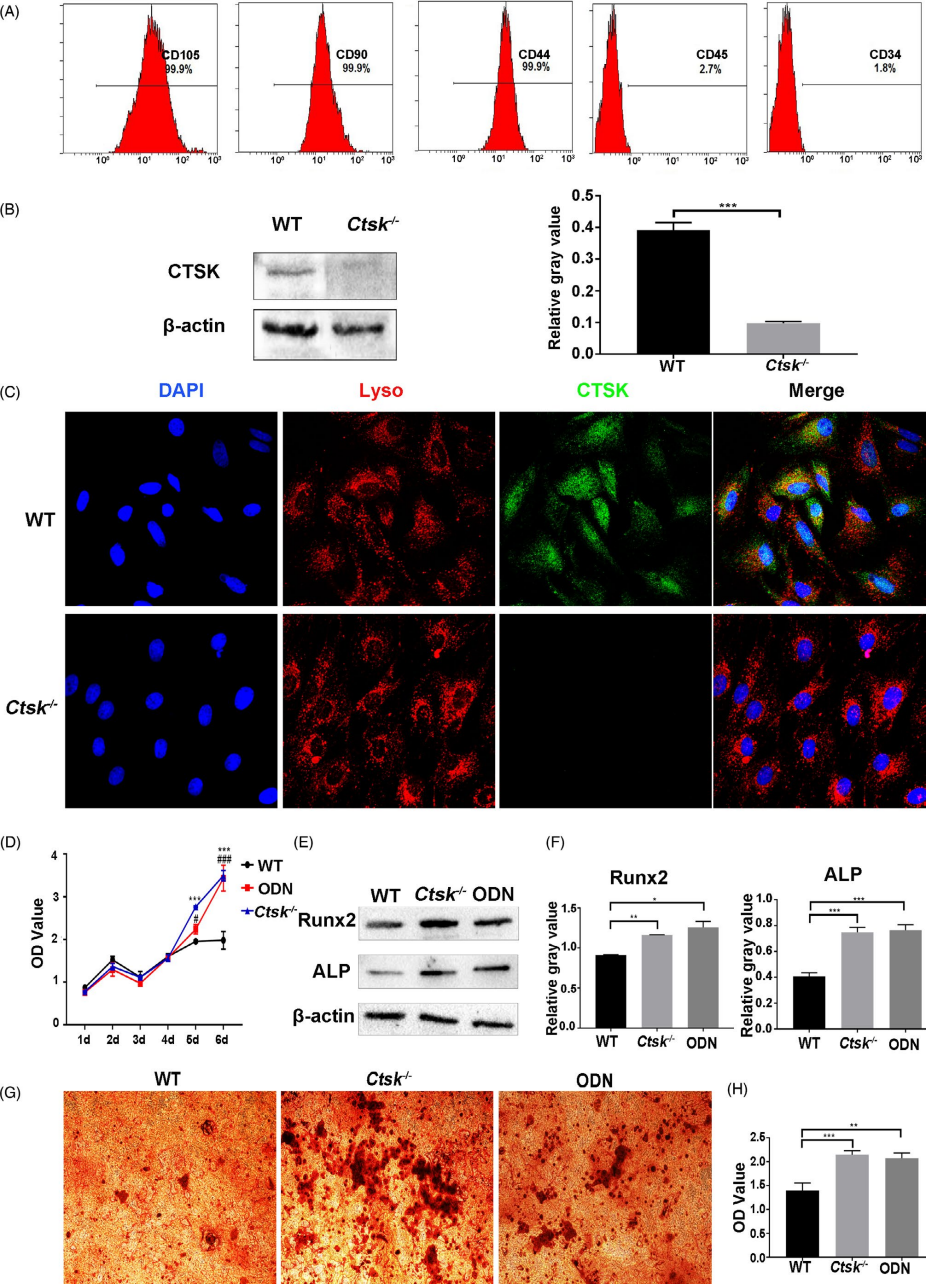
Endogenous *Ctsk* deficiency promotes JBMMSC proliferation and osteogenic differentiation. JBMMSC from *Ctsk^‐/‐^* mice and their WT littermates were cultured. A, Flow cytometry was used to detect the expression of JBMMSC surface markers. B, The expression of endogenous CTSK in JBMMSC and its deficiency in JBMMSC from *Ctsk* ^‐/‐^ mice were confirmed by Western blot. C, CTSK was mainly located in lysosomal in WT JBMMSC by immunofluorescence. D, Influence of *Ctsk* knockout or inhibition by ODN on proliferation of JBMMSC was assessed by CCK‐8 (* represent WT compared to *Ctsk^‐/‐^* group; # represent WT compared to ODN group). E, Expressions of osteogenic‐related proteins of ALP and Runx2 were detected by Western blot after osteogenic induction for 7 d, and quantitative analyses were shown in (F). G, Mineralized nodules of JBMMSC were assayed by alizarin red staining after osteogenic induction for 15 d. H, Alizarin red staining was quantified with a spectrophotometer after dissolving by 10% cetylpyridinium chloride. The statistical analysis was shown: *P < .05;**P < .01; ***P < .001; #*P* < .05; ###*P* < .001

Jaw bone marrow mesenchymal stem cells from *Ctsk* ^‐/‐^ and WT mice were used to determine the influences of *Ctsk* deletion or inhibition on their biological characteristics. Firstly, JBMMSC were positive (>95%) for MSC surface markers (CD105, CD90, CD44) and negative for hematopoietic surface markers (CD45, CD34) (Figure 4A). And the expression of endogenous CTSK in JBMMSC and its deficiency in JBMMSC from *Ctsk* ^‐/‐^ mice were confirmed by Western blot (Figure 4B). Then, the location of CTSK in WT JBMMSC was determined by immunofluorescence. The result indicated that CTSK was mainly expressed in lysosomes, but also in cytoplasm (Figure 4C). Further research indicated that both knocking out *Ctsk* and inhibiting its activity with ODN could promote the proliferation of JBMMSC (Figure 4D), the expression of ALP and Runx2 (Figure 4E,F), as well as the matrix mineralization (Figure 4G,H). In other words, endogenous *Ctsk* deficiency promotes JBMMSC proliferation and osteogenic differentiation.

### 3.5 *Ctsk* deficiency or inhibition promotes JBMMSC proliferation and osteogenic differentiation by up‐regulating glycolysis

**FIGURE 5:**
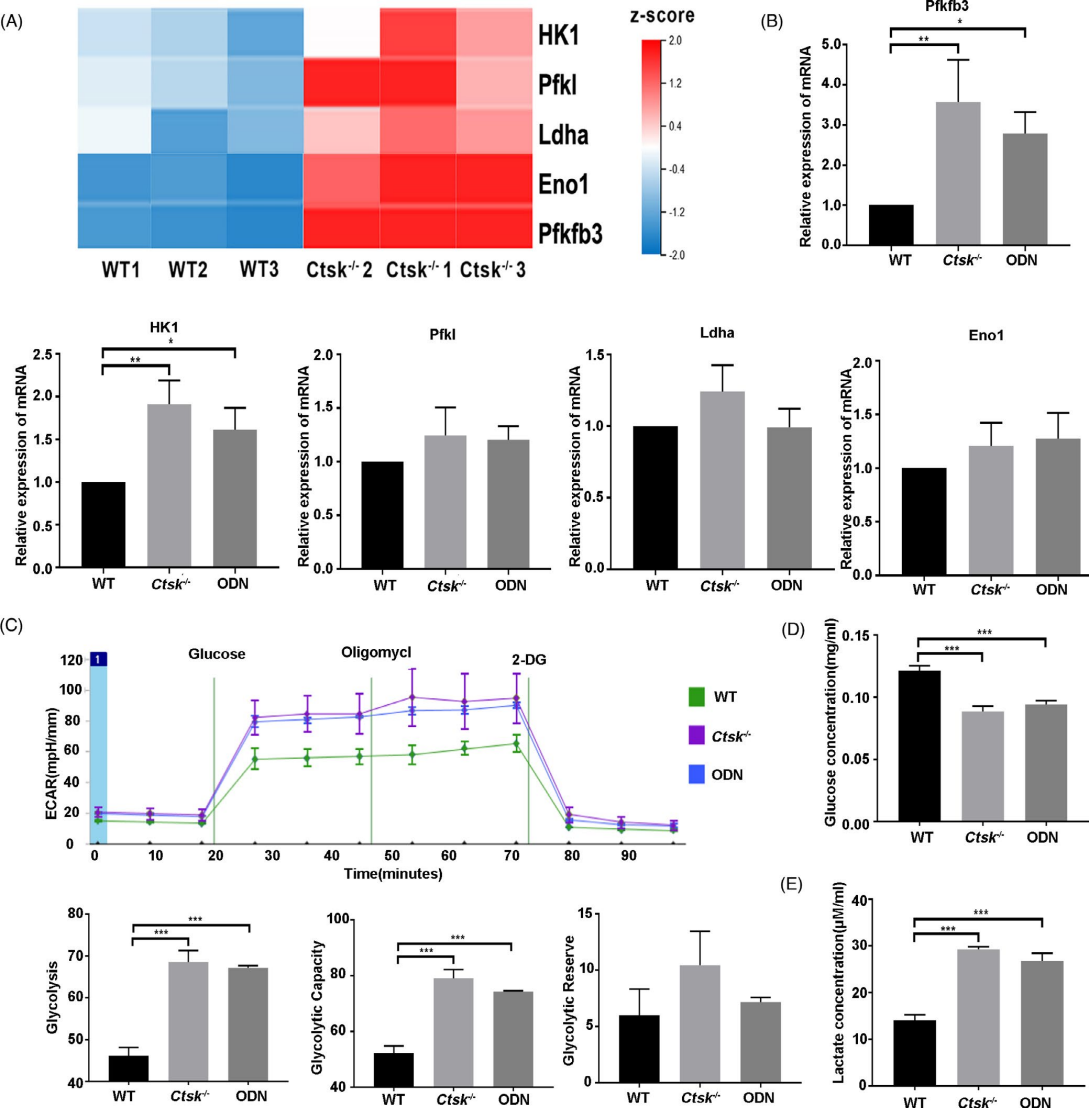
*Ctsk* deficiency or inhibition promotes glycolysis. JBMMSC from *Ctsk^‐/‐^* mice and their WT littermates were cultured and differentially expressed genes were selected by RNA‐seq. A, Five differentially expressed genes of key enzymes in glycolysis were detected by RNA‐seq. B, Expression levels of the five differentially expressed genes were confirmed by RT‐qPCR. C, WT and *Ctsk^‐/‐^*JBMMSC were stimulated with 1 μmol/L ODN or 1 μmol/L DMSO for 48 h and then detected extracellular acidification rate by Seahorse. Glucose consumption (D) and lactate production (E) were performed to study the effect of *Ctsk* deficiency or inhibition on glycolysis. The statistical analysis was shown: *P < .05; **P < .01; ***P < .001

In order to explore the possible mechanism underlying CTSK regulating the regeneration of JBMMSC, RNA‐seq was carried out in JBMMSC from WT and *Ctsk* ^‐/‐^ mice. Bioinformatics analysis results revealed that coding genes of key enzymes in glycolysis, including Pfkfb3, HK1, Pfkl, Eno1 and Ldha, were significantly up‐regulated in *Ctsk^‐/‐^* JBMMSC (FC > 1.2，*P* < .05) (Figure 5A). The increased expressions of Pfkfb3, HK1, Pfkl, Eno1 and Ldha were also confirmed by RT‐qPCR (Figure 5B). Additionally, there were 80 differentially expressed genes related to ATP synthesis, glycolysis, tricarboxylic acid cycle and ROS production between the two groups (FC ≥ 1.2, *P* < .05). Further analysis showed that HK1, Pfkfb3, Pfkl and Eno1 were the key driving genes in changing mitochondrial metabolism related genes (Figure S3).

Subsequently, we confirmed that *Ctsk* deficiency or inhibition by ODN could promote the glycolysis of JBMMSC. According to the results of ECAR, *Ctsk* deficiency or inhibition could significantly enhance the glycolysis and glycolysis capacity of JBMMSC (*P* < .05) (Figure 5C). Glucose consumption measurements showed that the concentration of glucose in the medium of *Ctsk^‐/‐^* or ODN group decreased significantly, indicating the increased glucose intake (*P* < .05) (Figure 5D). At the same time, increased lactate production was also detected in *Ctsk^‐/‐^* or ODN group (*P* < .05) (Figure 5E).

**FIGURE 6:**
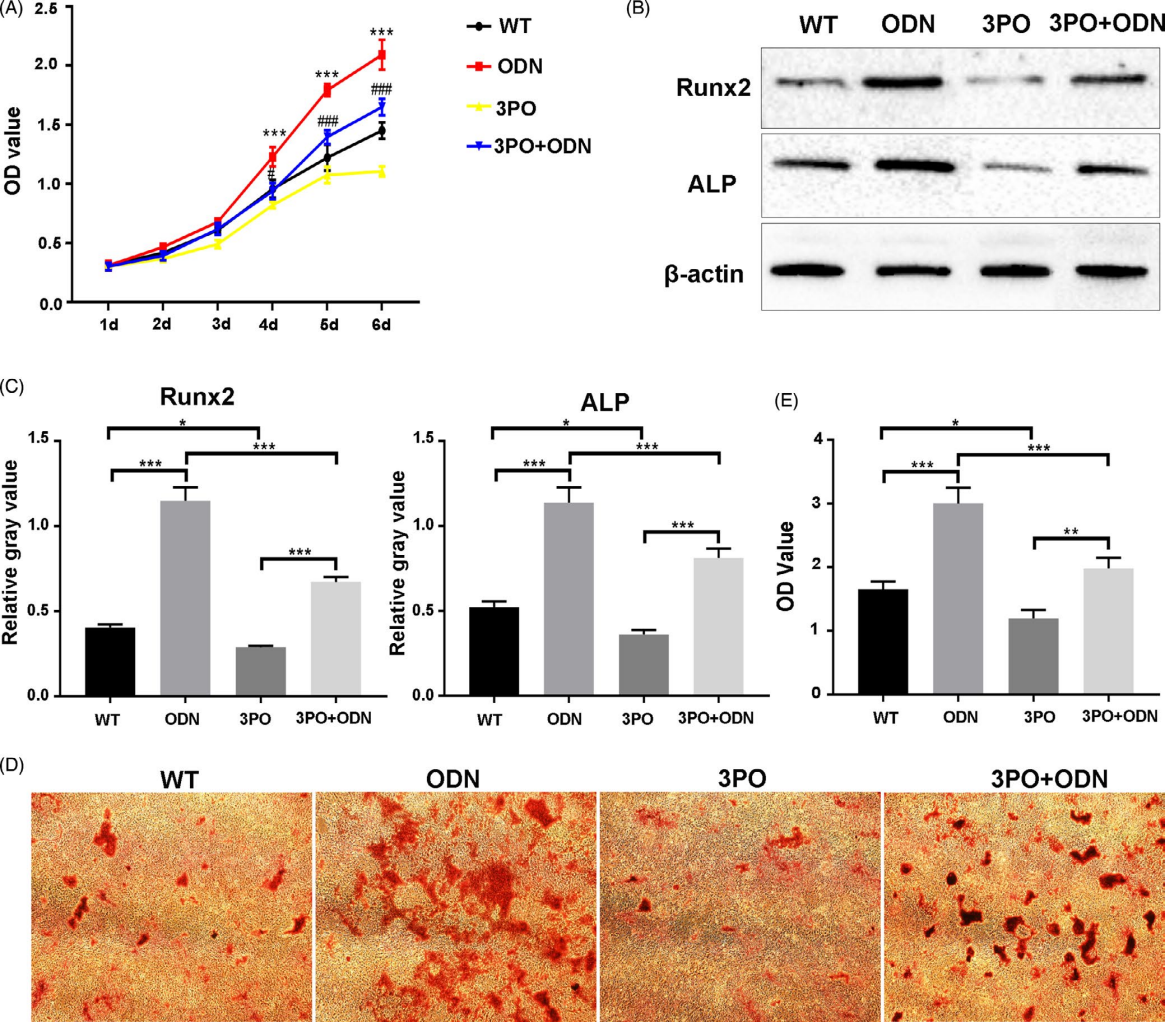
*C* *tsk* deficiency or inhibition promotes JBMMSC proliferation and osteogenic differentiation by up‐regulating glycolysis. JBMMSC from WT mice were stimulated with 1 μmol/L DMSO, 1 μmol/L ODN, 10 μmol/L 3PO or 1 μmol/L ODN + 10 μmol/L 3PO. A, Blocking glycolysis by 3PO inhibited the effect of ODN on JBMMSC proliferation (* represent WT compared to ODN group; # represent ODN compared to 3PO+ODN group). B, Expressions of osteogenic‐related proteins of ALP and Runx2 were detected by Western blot after osteogenic induction and chemical reagent treatment for 7 d. C, Quantitative analysis of Western blot images. D, Representative images of alizarin red staining of JBMMSC after osteogenic induction and chemical reagent treatment for 15 d and quantitative analyses were shown (E). The statistical analysis was shown: *P < .05; **P < .01; ***P < .001; #*P* < .05; ###*P* < .001

To confirm *Ctsk* deficiency or inhibition promoted JBMMSC proliferation and osteogenic differentiation by up‐regulating glycolysis, 3PO (a specific inhibitor of Pfkfb3) was used to inhibit glycolysis in *Ctsk^‐/‐^* or ODN treated JBMMSC. As a result, the ability of ODN or *Ctsk* deficiency to promote the proliferation of JBMMSC was blocked (*P* < .05) by 3PO (Figure 6A and Figure S4A). On the other hand, compared with the *Ctsk^‐/‐^* or ODN treated JBMMSC, the expression of Runx2 and ALP (Figure 6B,C and Figure S4B,C) as well as the mineral node formation (Figure 6D,E and Figure S4D,E) decreased in 3PO+ODN or *Ctsk^‐/‐^* +3PO group (*P* < .05). These results suggested that *Ctsk* deficiency or inhibition could promote JBMMSC proliferation and osteogenic differentiation by up‐regulating glycolysis.

## 4 DISCUSSION

The main findings of the present study include (a) *Ctsk* deficiency could regulate alveolar bone regeneration by promoting JBMMSC proliferation and differentiation; (b) *Ctsk* deficiency or inhibition promotes JBMMSC proliferation and osteogenic differentiation by up‐regulating glycolysis.

As a key regulator of bone metabolism, CTSK can regulate both bone resorption^1^, ^2^ and bone immunity.^15^, ^16^ So more and more studies focus on the dual regulation of CTSK on bone immunity and bone remodelling.^17^, ^18^, ^19^ Silencing of *Ctsk* by AAV‐RNAi was used to inhibit the development of periapical periodontitis in mice.^20^ Silencing of *Ctsk* or inhibiting the function of CTSK by ODN was also used to inhibit inflammation and bone loss caused by periodontal diseases.^17^, ^21^ These studies confirmed the preventive effect of *Ctsk* deficiency/inhibition on alveolar bone resorption secondary to odontogenic inflammatory diseases. However, whether *Ctsk* deficiency/inhibition could promote alveolar bone regeneration and then be used to treat alveolar bone defects remains unclear. In this study, tooth extraction models were used to investigate the role of CTSK in alveolar bone regeneration. As a result, we found that *Ctsk* knockout could significantly promote the new bone formation in the early stage of the socket healing and accelerate the regeneration of alveolar bone. During this period, neither the number nor the morphology of osteoclasts showed significant difference between *Ctsk^‐/‐^* and WT mice. Contrarily, Osx‐positive cells in *Ctsk^‐/‐^* mice peaked earlier. The aim of this study is not to overturn the classical theory that CTSK regulates bone regeneration through osteoclasts. However, the fact that CTSK is expressed not only in osteoclasts but also osteoblast cells during the healing process after tooth extraction suggests that there may be other pathways for CTSK regulating bone regeneration. In other words, the regulation of CTSK on early alveolar bone healing and regeneration may depend on JBMMSC mediated bone formation.

It has been reported that CTSK can indirectly regulate bone formation by regulating osteoclasts. Briefly, inhibition of CTSK could increase the number of preosteoclasts and the endogenous levels of platelet‐derived growth factor‐BB, which could increase CD31(hi)Emcn(hi) vessel number and stimulate BMMSC proliferation and migration in mice.^22^, ^23^ However, CTSK was also expressed in osteoblasts from different bone^24^, ^25^ and different kinds of MSC.^3^, ^4^, ^5^ The function of endogenous CTSK in these cells remains unclear. Using CTSK knockdown experiments, Whitty et al^4^ showed that CTSK actively controlled sclerostin levels, subsequently affected Wnt/β‐catenin pathway in PDL fibroblasts through a lysosomal mechanism. These results indicated that endogenous CTSK in these cells may be related to osteogenic activity. In this study, we confirmed the expression and location of CTSK in JBMMSC both in vitro and in vivo, and revealed that knocking out *Ctsk* or inhibiting its activity with ODN could promote the proliferation and osteogenic differentiation of JBMMSC in vitro.

In order to detect how CTSK regulates the regeneration of JBMMSC, RNA‐seq was carried out in JBMMSC from WT and *Ctsk* ^‐/‐^ mice. Bioinformatics analysis indicated the effect of CTSK on glycolysis. Glycolysis is the common stage of all the three pathways for the oxidative decomposition of glucose, including anaerobic oxidation, aerobic oxidation and pentose phosphate pathway. It has been reported that cells in proliferative tissues tend to convert up to 85% glucose to lactate regardless of whether oxygen is present.^26^ It has been confirmed that glycolysis plays an important role in regulating the proliferation and osteogenic differentiation of BMMSC.^10^, ^11^ However, there are few reports on the role of CTSK on glycolysis.

There are a few studies on the relationship between CTSK and glucose metabolism. Yang et al^12^ reported that *Ctsk* deficiency/inhibition could enhance glucose metabolism in adipose tissue and block the adipogenic differentiation of 3T3‐L1 cells. But Dauth et al^13^ found that *Ctsk* knockout did not affect the morphology and glucose metabolism of astrocytes. Recently, it was reported that selective inhibition of CTSK by ODN could increase the production of ROS in mitochondria of human renal carcinoma Caki cells.^14^ In this study, we not only confirmed that *Ctsk* deficiency/inhibition could promote the glycolysis of JBMMSC, but also revealed that *Ctsk* deficiency/inhibition promoted JBMMSC proliferation and osteogenic differentiation by up‐regulating glycolysis.

In summary, we have demonstrated that both knockout and inhibition of CTSK could promote JBMMSC proliferation and osteogenic differentiation by up‐regulating glycolysis, thereby promote alveolar bone regeneration. Though these results could provide some evidence for promoting the regeneration of JBMMSC by inhibiting CTSK for jaw bone regeneration in different environments, how CTSK regulate glycolysis needs further research, and mice with conditional knockout of CTSK in BMMSC should be generated, too.

## CONFLICT OF INTEREST

The authors declare no competing interests regarding the publication of this paper.

## AUTHOR CONTRIBUTIONS

Authors’ roles: Study design: YX, KH, HZ, WZ and ZD; Study conduct: WZ, ZD, DL and HL; Data collection: WZ, ZD, DL and XZ; Data analysis: WZ, ZD, DL, BL, YL and XZ; Manuscript preparation: YX, WZ and ZD; Revising manuscript content: YX, KH, HZ, WZ and ZD.

## Supporting information

Supplementary Material

## Data Availability

The data that support the findings of this study are available from the corresponding author upon reasonable request.
