# Supplementary material for: Cathepsin K deficiency promotes alveolar bone regeneration by promoting jaw bone marrow mesenchymal stem cells proliferation and differentiation via glycolysis pathway
Source: Cell Prolif. 2021 May 30;54(7):e13058. doi: 10.1111/cpr.13058 (PMC8249792; doi:10.1111/cpr.13058)
Supplement: Supplementary file 1 — Supplementary Material [file CPR-54-e13058-s001.docx]

**
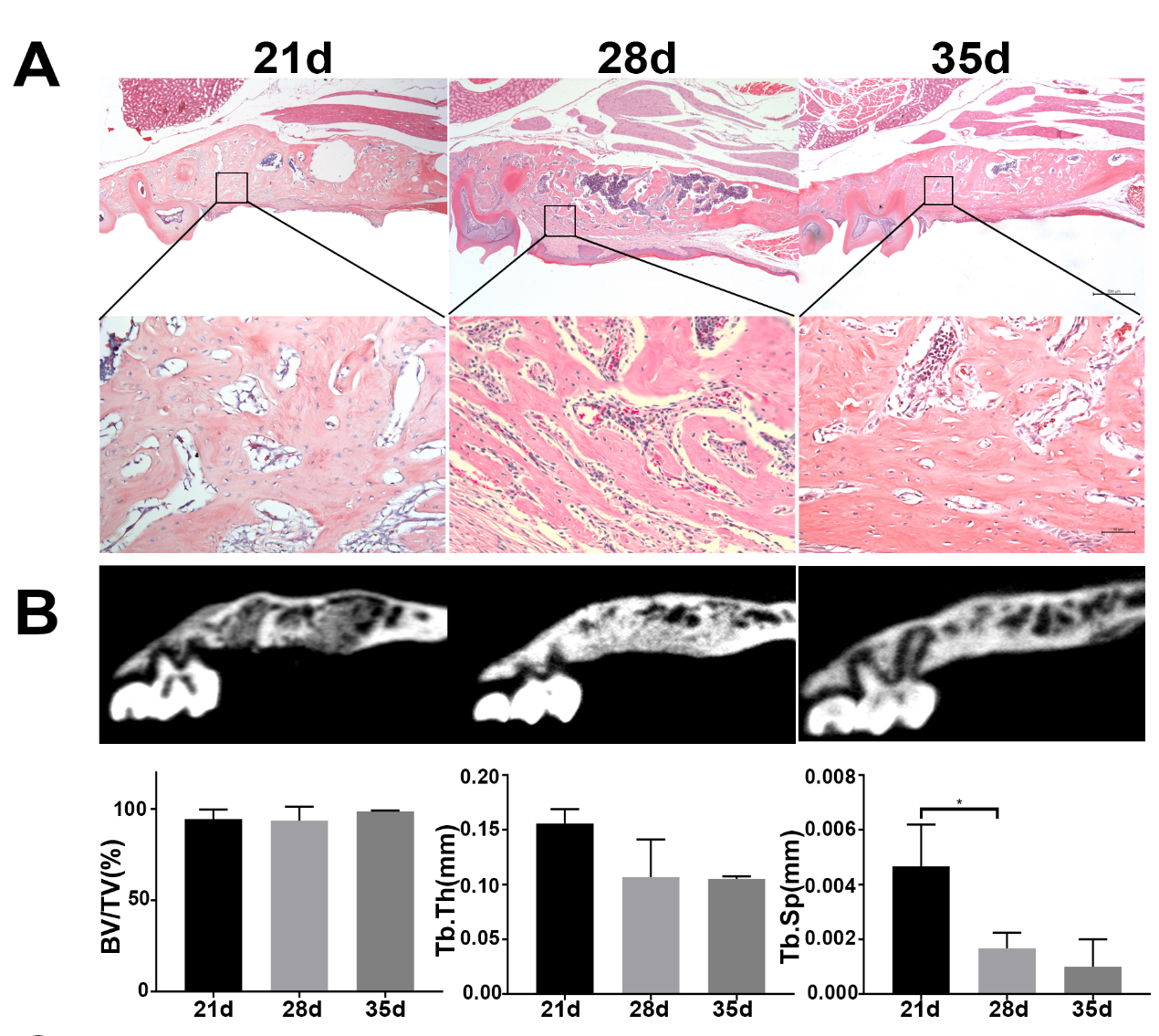
**

**Suppl. Fig. 1 Late process of bone remodeling after tooth extraction in mice.** There are two processes after tooth extraction, including bone filling and remodeling. In mice, the bone filling stage was during the first 14 days, while the bone remodeling stage began with the onset of bone filling and lasted for 35 days. (A) Representative images of H&E staining results at 21 d, 28 d, 35 d after extraction extraction. (B) Representative Micro-CT scanning images of the extraction socket and quantitative analysis of the morphological parameters of trabecular bone. The statistical analysis was shown: *P < 0.05; **P < 0.01.


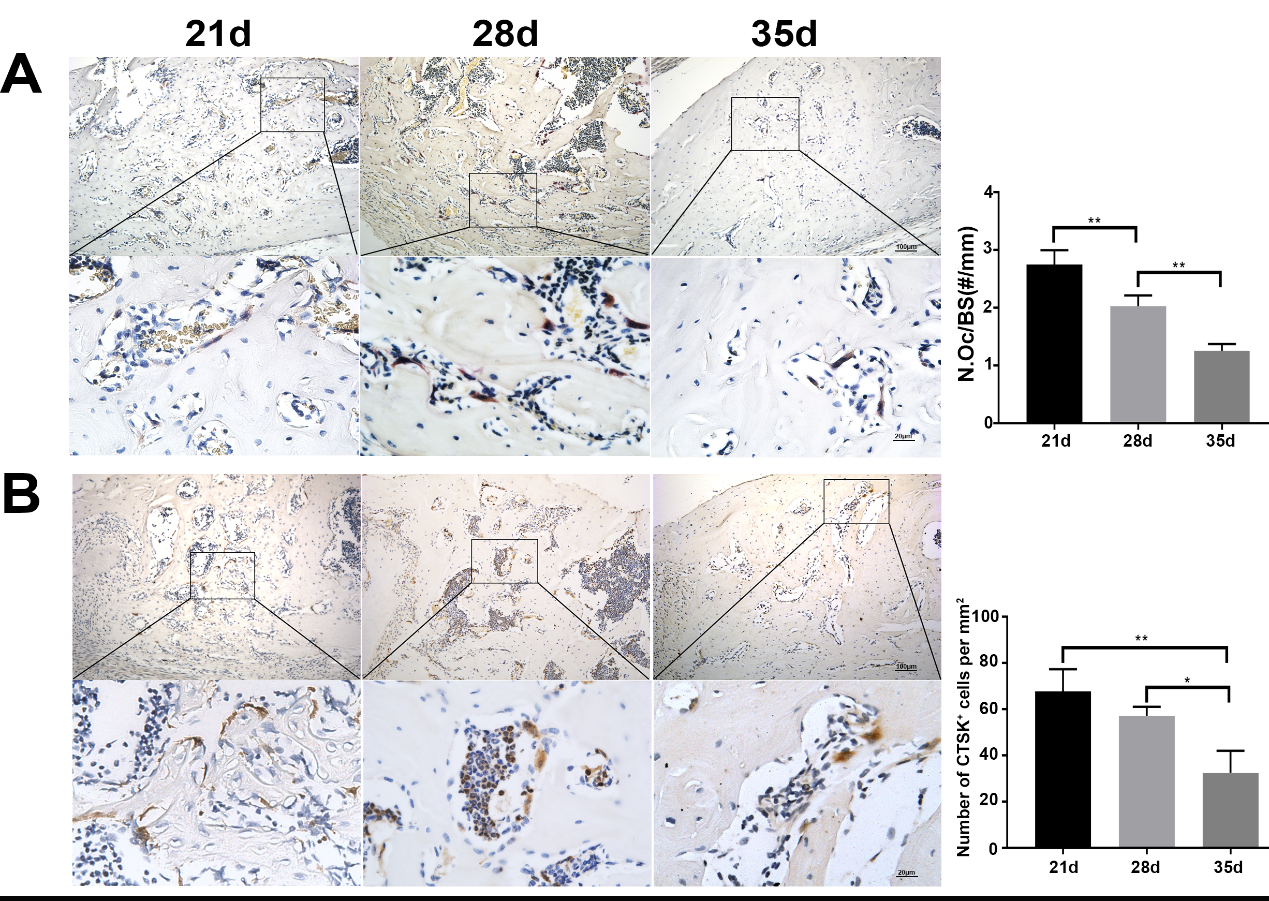


**Suppl. Fig. 2 Distribution of TRAP+ cells and CTSK+ cells during the late process of bone remodeling.** (A) Representative images of TRAP staining and the quantitative analysis. (B) Immunohistochemistry staining and quantitative analysis of CTSK. The statistical analysis was shown: *P < 0.05; **P < 0.01.


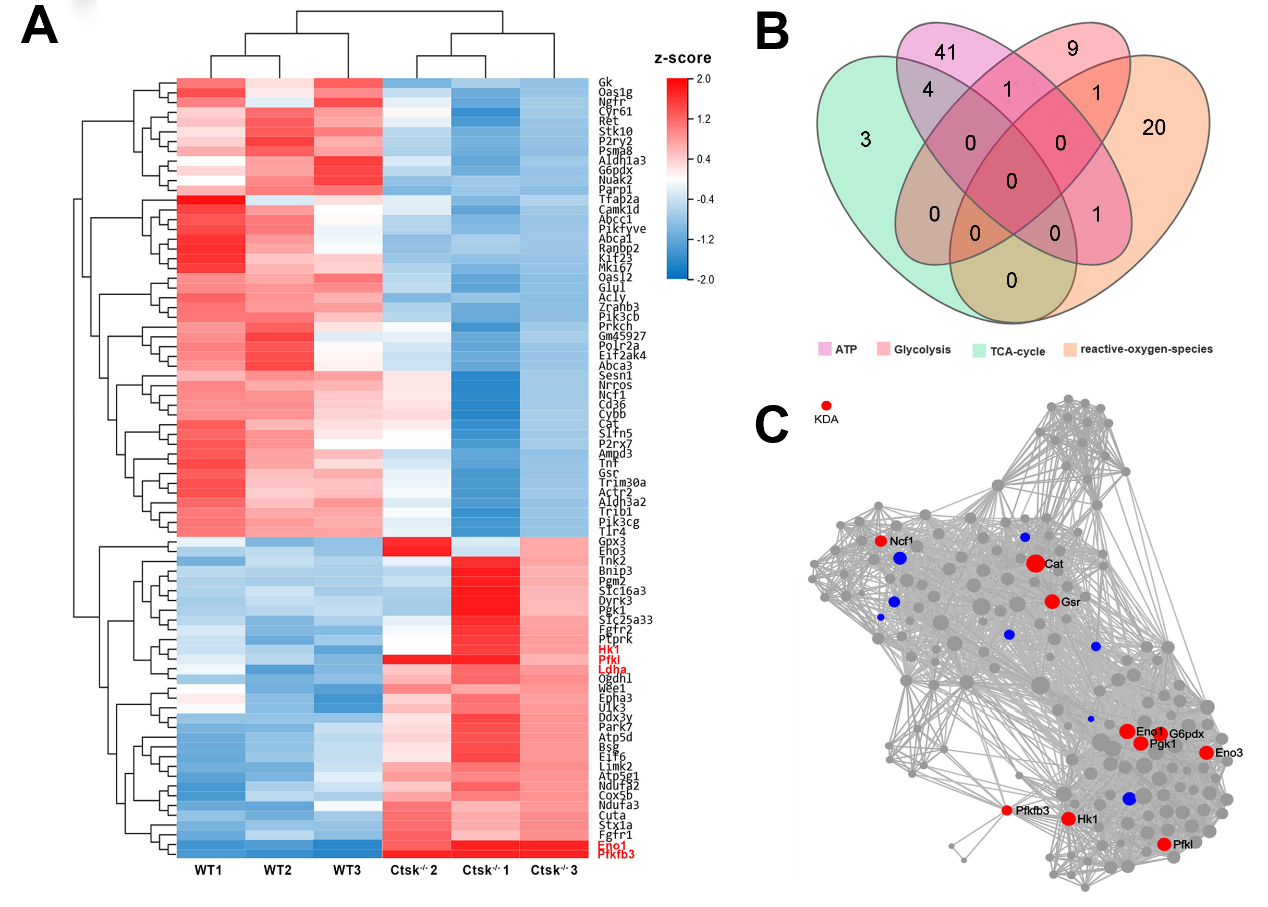


**Suppl. Fig. 3 Bioinformatics analysis results of RNA-seq.** In order to explore the possible mechanism underlying CTSK regulating the regeneration of JBMMSC, RNA-seq was carried out in JBMMSC from WT and *Ctsk*^-/-^ mice. (A) There were 80 differentially expressed genes (FC≥1.2, P<0.05).related to ATP synthesis, glycolysis, tricarboxylic acid cycle (TAC), and reactive oxygen species (ROS) production between the two groups. (B) Among the 80 differentially expressed genes, there were 47 genes related to ATP synthesis, 11 genes related to glycolysis,7 genes related to TAC and 22 genes related to ROS production. (C) Further analysis showed that HK1, Pfkfb3, Pfkl and Eno1 were the key driving genes in changing metabolism related genes.

**
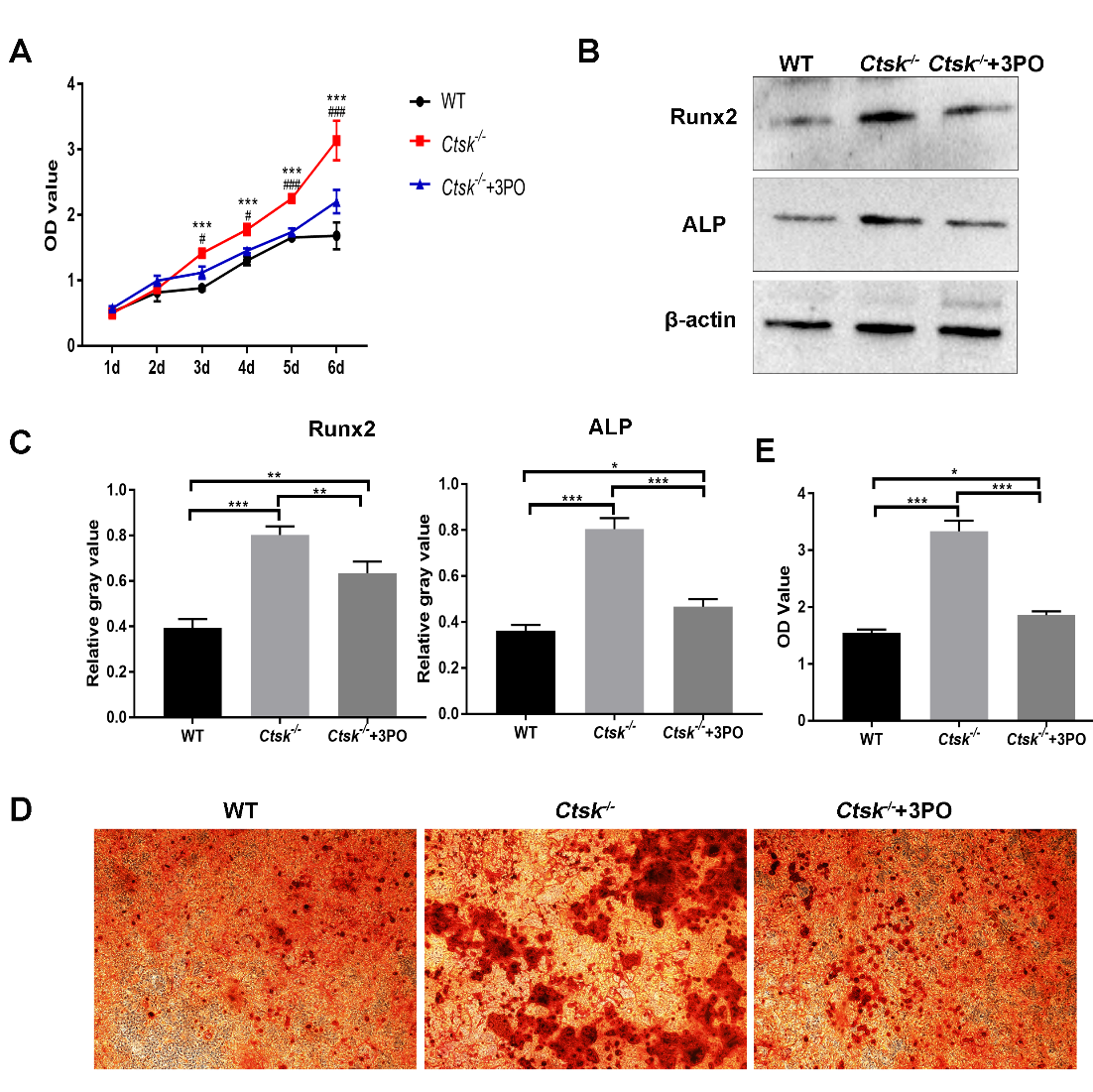
**

**Suppl. Fig4. 3PO can rescue the phenotype of *Ctsk^-/-^* cells.** JBMMSC from *Ctsk^-/-^* mice were stimulated with 10 μM 3PO and JBMMSC from WT and *Ctsk^-/-^* mice were stimulated with 1 μM DMSO. (A) Blocking glycolysis by 3PO inhibited the effect of *Ctsk* knockout on JBMMSC proliferation (* represent WT compared to *Ctsk^-/-^* group; # represent 3PO compared to *Ctsk^-/-^* group). (B) Expressions of osteogenic-related proteins of ALP and Runx2 were detected by Western blot after osteogenic induction for 7 days. (C) Quantitative analysis of Western blot images. (D) Representative images of alizarin red staining of JBMMSC after osteogenic induction for 14 days and quantitative analyses were shown (E). The statistical analysis was shown: **P* < 0.05; ***P* < 0.01; ****P* < 0.001; ###*P* < 0.001.

**Suppl. Table. 1** Sequence of primers for RT-qPCR analysis

| Gene name | Forward | Reverse |
| --- | --- | --- |
| Pfkfb3 | GATCTGGGTGCCCGTCGATCACCG | CAGTTGAGGTAGCGAGTCAGCTTC |
| HK1 | CACGTGGACAAAGGGATTCA | CCACCACATCCAGGTCAAAT |
| Ldha | CAGACTTGGCTGAGAGCATAA | GATACATGGGACACTGAGGAAG |
| Pfkl | GGGTCATGTACAGCGAGGA | GGCCTCCATACCCATCTTG |
| Eno1 | AGCGATCCTACTGCCAGAAAT | GATCGACCTCAACAGTGGGA |
| β-actin | CCACTGCCG CATCCTCTTCC | CTCGTTGCCAATAGTGATGACCTG |
